# Very small embryonic-like stem cells (VSELs) on the way for potential applications in regenerative medicine

**DOI:** 10.3389/fbioe.2025.1564964

**Published:** 2025-03-07

**Authors:** Kannathasan Thetchinamoorthy, Justyna Jarczak, Patrycja Kieszek, Diana Wierzbicka, Janina Ratajczak, Magdalena Kucia, Mariusz Z. Ratajczak

**Affiliations:** ^1^ Laboratory of Regenerative Medicine, Medical University of Warsaw, Warsaw, Poland; ^2^ Stem Cell Institute at Graham Brown Cancer Center, University of Louisville, Louisville, CO, United States

**Keywords:** pluripotent stem cells, tissue organ regeneration, very small embryonic-like stem cells (VSELs), embryonic stem cells, induced pluripotent stem cells

## Abstract

Evidence has accumulated that adult tissues contain a population of early development stem cells capable of differentiating across germ layers into various types of cells. Our group purified these rare cells, naming them very small embryonic-like stem cells (VSELs). With their broad differentiation potential, VSELs have emerged as a new candidate population for clinical applications. This advancement is now possible due to our recent development of a model for *ex vivo* expansion of these rare cells. Importantly, no evidence suggests that VSELs, isolated from adult tissues, can form teratomas. In this review paper, we update current research on these cells reported in our laboratory as well as in those of several independent investigators.

## Introduction

Regenerative medicine, an exciting and rapidly evolving field of biomedical science, is founded on two key concepts. First, it involves the use of stem and progenitor cells, either alone or in combination with organic or synthetic scaffolds, to regenerate or even replace damaged tissues. Second, it aims to develop strategies that enhance the robustness of adult stem cells found in various organs and tissues, ensuring proper regeneration and healthy aging (Suman et al., 2019). The primary goal remains the identification of pluripotent or multipotent stem cells capable of differentiating across germ layers, which can be safely utilized in therapy. Unfortunately, aside from hematopoietic stem cells (HSCs) isolated from the bone marrow (BM), which have been used for 60 years in hematological transplants, current clinical outcomes with stem cells for regenerating other organs are significantly lagging (Suman et al., 2019; Balassa et al., 2019). In addition to BM, HSCs are more commonly isolated from mobilized peripheral blood (mPB) and umbilical cord blood (UCB). The successful clinical application of HSCs has provided promise and justification for advancing stem cell-based therapies in regenerative medicine. However, the monopotency and limited differentiation potential of HSCs pose challenges for their clinical use in repairing non-hematopoietic organs.

This is why the extensive search for pluripotent and multipotent stem cells capable of differentiating across germ layers began. Potential candidates include embryonic stem cells (ESCs) isolated from embryos (Damdimopoulou et al., 2016) and induced pluripotent stem cells (iPSCs) generated through the genetic manipulation of somatic cells (Shi et al., 2017). Unfortunately, the anticipated clinical applications of these cells have been somewhat disappointing, as both types present various challenges, such as genomic instability, the risk of teratoma formation, and the potential for rejection of these cells and their progeny (Yoshihara et al., 2017; Tapia and Scholer, 2016; Deuse et al., 2015; Bhartiya, 2018).

However, recent reports on the use of chemically induced pluripotent stem cells are promising. For instance, chemically iPSC-derived islets in a patient with type 1 diabetes resulted in acceptable safety and encouraging restoration of exogenous insulin independence, with glycemic control observed at the 1-year follow-up (Wang et al., 2024). Additionally, there were no serious adverse events, tumors, or other safety concerns associated with the systemic delivery of iPSC-derived mesenchymal stem cells for treating chronic graft-versus-host disease. This delivery method proved safe and well-tolerated over 2 years of follow-up, with sustained outcomes maintained up to 2 years after the initial infusion (Kelly et al., 2024). Therefore, we anticipate exciting advancements in the application of iPSCs in the years ahead (Ramzy et al., 2021).

An alternative method for obtaining PSCs has been proposed to avoid rejection and provide histocompatible cells derived from ESCs produced through the nuclear transfer strategy (Gouveia et al., 2020). However, these cells also carry the risk of teratoma formation (Suman et al., 2019; Zhao et al., 2011). Additionally, even if the donor nucleus contains the donor’s histocompatibility antigens in the nuclear transfer strategy, these cells still face the risk of rejection due to mitochondrial antigens inherited from the oocyte (Deuse et al., 2015). Finally, deriving fully functional cells (e.g., hematopoietic cells) from ESCs and iPSCs remains a challenge (Rao et al., 2022). This highlights a significant barrier to the clinical application of ESCs and iPSCs unless we can control their fate and ensure efficient tissue specification.

What is essential for this review series published in the “*Frontiers in Bioengineering and Biotechnology*” journal is that solid results indicate adult tissues contain rare, early-development stem cells that can potentially differentiate into cells from multiple germ layers (D'Ippolito et al., 2004; Beltrami et al., 2007; Wakao et al., 2018; Alanazi et al., 2023; Orlic et al., 1994; Jones et al., 1990; Krause et al., 2001; Vacanti et al., 2001; Suszynska et al., 2014; Kucia et al., 2006). These scarce cells often “contaminate” preparations of cells isolated from adult tissues and have been labeled with various names based on the strategies used for their identification. These cells have been referred to as: i) marrow-isolated adult multilineage-inducible (MIAMI) cells (D'Ippolito et al., 2004), ii) multipotent adult stem cells (MASCs) (Beltrami et al., 2007), iii) multilineage-differentiating stress-enduring cells (Muse) (Wakao et al., 2018; Alanazi et al., 2023), iv) elutriation-derived, Lin–after BM homing-isolated (ELH) cells (Orlic et al., 1994; Jones et al., 1990; Krause et al., 2001), v) spore-like stem cells (Vacanti et al., 2001), and vi) very small embryonic-like stem cells (VSELs) (Jones et al., 1990; Krause et al., 2001). It is conceivable that all these stem cells, some of which are poorly characterized at the single-cell level and described by different investigators, are closely related and represent overlapping cell populations, even if they vary in levels of specification (Suszynska et al., 2014). The best characterized at the single-cell level so far are VSELs (Kucia et al., 2006; Kucia et al., 2007; Jarczak et al., 2024) and Muse cells (Wakao et al., 2018; Alanazi et al., 2023). Furthermore, a recent elegant paper demonstrated that ELH cells correspond to VSELs (Kassmer et al., 2013; Ciechanowicz et al., 2021). In this updated review, we will focus on VSELs, which were discovered 20 years ago by our team. Based on our results and data from independent research groups, we will summarize and update the approach that led to their discovery and the current status of VSEL research. Due to space limitations, we will discuss the most relevant data, and all other studies from independent groups that described VSELs or small cells expressing VSEL markers and morphology are listed in Supplementary Table 1.

### Relationship of VSELs to BM small cells, mystery population of stem cells, ELH cells, and muse cells

VSELs are very small cells, comparable in size to those found in the inner cell mass of the blastocyst. Depending on the measurement method (in suspension or after adherence to slides), they measure approximately 3–5 µm in mice and 5–6 μm in humans (Suszynska et al., 2014; Kucia et al., 2006; Kucia et al., 2007). Because they are slightly smaller than red blood cells, a unique gating strategy is necessary during FACS sorting. Transmission electron microscopy analysis has shown that VSELs possess large nuclei filled with euchromatin and a thin rim of cytoplasm rich in spherical mitochondria, a characteristic marker of early developmental germline cells (Kucia et al., 2006; Kucia et al., 2007). Below, we will discuss the types of very small cells identified in the past within hematopoietic tissues that may correspond to the already specified VSELs.

- *BM small cells.* The detection of small cells in the BM has a long history; however, in many instances, these cells were not well characterized at the molecular level and were often assumed to be part of the hematopoietic lineage. This research began 35 years ago when Matsuoka et al. employed an electron microscope to describe cells among the BM mononuclear cells measuring between 4 and 5 μm, which displayed morphological features characteristic of hematopoietic progenitors (Matsuoka and Tavassoli, 1989). Subsequently, Radley et al. described long-term repopulating HSCs in the murine BM Lin^−^/Rh^dull^/Ho^dull^, averaging approximately 4–5 µm in size (Radley et al., 1999). Following these discoveries, Jones et al. isolated small cells from murine BM measuring <5 ∝m as a population of long-term repopulating HSCs. These cells were purified using elutriation followed by FACS-based high aldehyde dehydrogenase activity (ALDH^high^) and a lack of hematopoietic lineage markers typical of more mature hematopoietic progenitors (Jones et al., 1996). They were named based on the isolation strategy that involved elutriation and antigen staining (fractionated (Fr25) via elutriation, lineage-depleted (lin^−^), and labeled with PKH26) or ELH stem cells. Recent seminal work by Krause et al. indicated that these cells correspond to VSELs (Kassmer et al., 2013; Ciechanowicz et al., 2021). Additional small cells isolated from the BM include a population of spore-like cells identified by Vacanti et al. (Vacanti et al., 2001) and very small CD45^−^Sca-1^+^c-kit^-^ cells isolated from murine BM by Howell et al. (Howell et al., 2003).
- *A “mystery” population of small CD45* ^*+*^ *stem cells exists in murine bone marrow (BM).* These small cells, exhibiting numerous phenotypic traits associated with resting hematopoietic stem cells (HSCs), were identified in response to Jones’ data by Randall & Weissman (Randall and Weissman, 1998). Nevertheless, these “mystery” cells were found to be quiescent, lacking hematopoietic specification in both *in vitro* and *in vivo* assays. Consequently, they share characteristics similar to freshly isolated BM very small embryonic-like stem cells (VSELs) (Kucia et al., 2006; Kucia et al., 2007). Notably, the overall size of the “mystery” population falls between that of VSELs and HSCs. Based on this observation, we hypothesized that following the purification of VSELs, the quiescent CD45^+^ “mystery” population could reside in BM tissue, representing a missing developmental link between VSELs and HSCs (Randall and Weissman, 1998; Bujko et al., 2023). To support this idea, when we conduct assays similar to those we routinely use to specify VSELs into HSCs, the cells from the “mystery” population specified over OP9 stroma were able to generate hematopoietic colonies and establish hematopoietic chimerism in lethally irradiated recipients. Based on these results, the murine BM “mystery” population may represent an intermediate population between BM-residing CD45^−^ VSELs and CD45^+^ HSCs (Bujko et al., 2023).
- *Multilineage-differentiating stress-enduring cells (Muse cells)* represent an intriguing population of stem cells that express stage-specific antigen 3 (SSEA3) on their surface, a well-known marker of undifferentiated human embryonic stem (ES) cells (Cancedda and Mastrogiacomo, 2024). They are distinct from other stem cell populations in bone marrow (BM) due to their size, measuring approximately 13–15 μm in diameter. As a result, they differ in size and surface marker expression from very small embryonic-like stem cells (VSELs), as they do not express CD34 or CD117 antigens (Wakao et al., 2018; Alanazi et al., 2023). Muse cells are found in the connective tissue of nearly every organ, including the BM, and can be isolated along with adherent cells from umbilical cord blood (UCB) and peripheral blood (PB) (Wakao et al., 2018; Alanazi et al., 2023). However, similar to VSELs, they can differentiate into cells from all three germ layers, express pluripotency genes, and do not form teratomas (Park et al., 2020). Further research is needed to explore the potential relationship with VSELs, as this could suggest that these cells are more specified derivatives of VSELs.

### From the concept of stem cell plasticity to the discovery of VSELs

As we mentioned above, the use of stem cells isolated from adult tissues in regenerative medicine was initially misled by the concept of “stem cell plasticity.” It was suggested that fully specified stem cells, taken from murine bone marrow and believed to be HSCs, could dedifferentiate into cells from non-hematopoietic lineages, such as cardiomyocytes. While some studies reported positive results regarding the enhancement of damaged tissue function, explaining this phenomenon proved to be challenging (Suman et al., 2019; Cancedda and Mastrogiacomo, 2024).

From the beginning, our team was skeptical of enthusiastic reports suggesting the transdifferentiation or plasticity of adult stem cells. Terminal differentiation occurs during stem cell specification and is irreversible in postnatal tissues. To explain some positive functional data observed after adult stem cell therapies, we proposed three potential explanations, supported by key observations from our laboratory.

**FIGURE 1:**
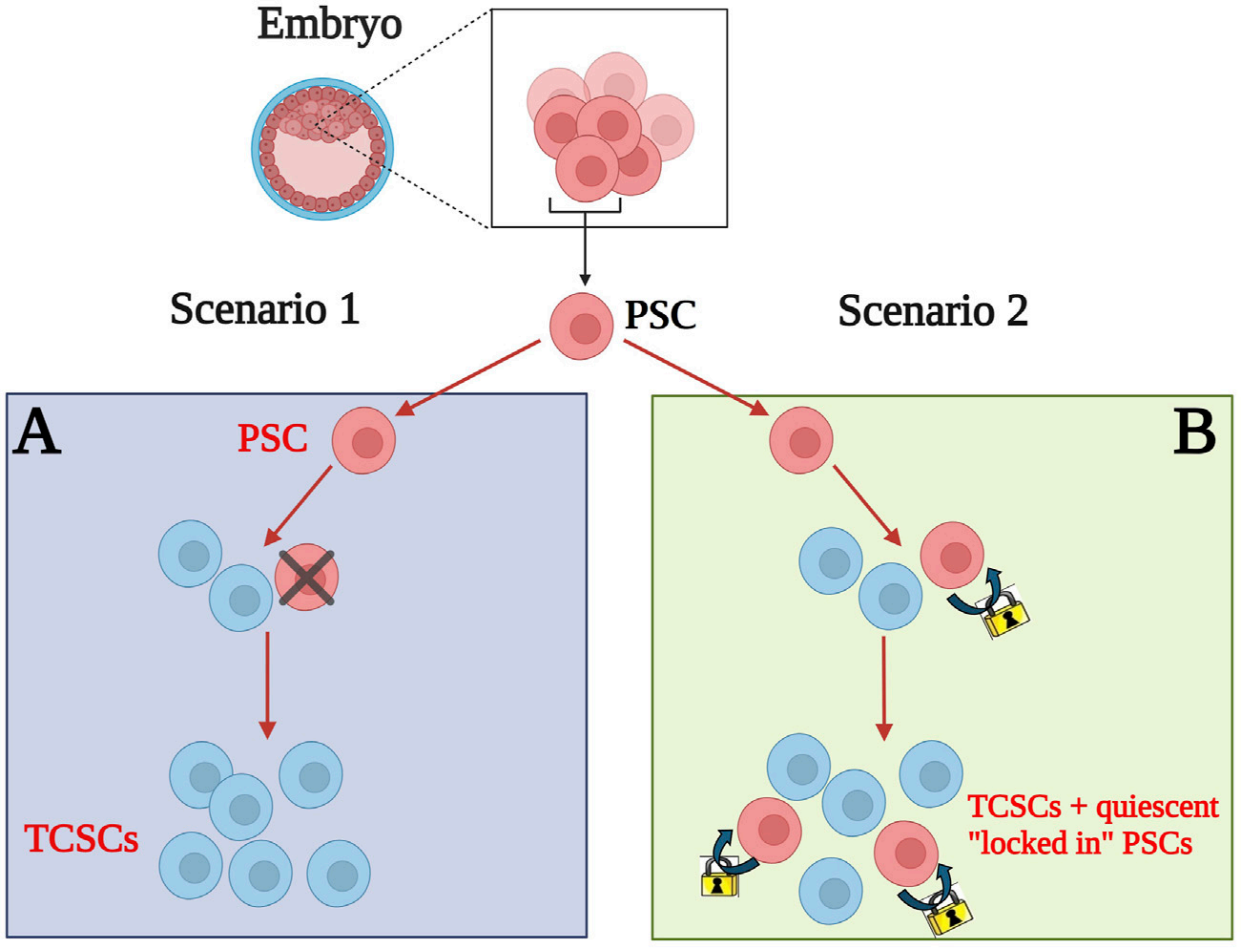
Two distinct developmental scenarios for the presence of pluripotent stem cells (PSCs) in postnatal tissues. **(A)** Scenario 1: PSCs in the inner cell mass of the blastocyst develop into monopotent tissue-committed stem cells (TCSCs) and subsequently vanish from the developing embryo. **(B)** Scenario 2: PSCs also give rise to TCSCs; however, some of these cells remain in postnatal tissues as a backup population for TCSCs. Nevertheless, effective molecular mechanisms involving the erasure of specific paternal imprinted genes prevent them from forming teratomas.

First, as we previously reported, human CD34^+^ HSCs express various growth factors, cytokines, chemokines, and bioactive lipids at the protein level as part of their secretome—all of which may prevent apoptosis, promote vascularization, and support the proliferation of tissue-committed stem cells in damaged organs. Therefore, we proposed that adult stem cell therapies relate to the paracrine effects of secreted bioactive factors (Majka et al., 2001; Ratajczak J. et al., 2013). Two decades ago, we also suggested that stem cells could transfer mRNA and proteins, thereby conveying potential anti-apoptotic and vasculogenic signals from 1 cell to another via extracellular microvesicles (Ratajczak et al., 2006). The larger microvesicles originate from the budding of the cell surface membrane, while smaller ones, known as exosomes, are released from the endosomal compartment. Interestingly, after an initial wave of skepticism, this significant cell-cell communication and pro-regenerative phenomenon was ultimately verified by other researchers and is clearly observable, for instance, after therapies involving mesenchymal stem cells (MSCs). It illustrates how novel scientific concepts sometimes require time to overcome initial disbelief (Ratajczak and Ratajczak, 2020). Lastly, we envisioned that some multipotent stem cells might persist in adult tissues, remaining dormant since embryogenesis, serving as a backup population for tissue-committed stem cells (Suszynska et al., 2014; Kucia et al., 2006; Kucia et al., 2007; Ratajczak et al., 2019; Ratajczak et al., 2004). Figure 1 depicts two scenarios regarding the fate of pluripotent stem cells during embryogenesis. In the first scenario (Figure 1A), pluripotent stem cells are located in the inner cell mass of the blastocyst, where they are specified into multipotent and subsequently monopotent stem cells throughout organ and tissue development, eventually disappearing from postnatal tissues. In the second scenario (Figure 1B), they remain within the tissues, contributing to the generation of monopotent stem cells, while some survive in a dormant state as a backup population of stem cells in developed organs. However, a regulatory mechanism must exist to keep them under control and prevent uncontrolled proliferation that could lead to tumors and teratomas. This mechanism, based on the erasure of paternal imprinting, will be presented and discussed later (Shin et al., 2009).

We began searching for rare small dormant pluripotent/multipotent stem cells in adult tissues. Our initial experiments revealed that these cells are indeed very small, comparable in size to those found in the inner cell mass of the blastocyst, and they express specific genes that regulate pluripotency. This led us to identify a population of small, early-development stem cells in adult tissues that exhibit pluripotency markers and, based on their primitive morphology and gene expression profile, were named very small embryonic-like stem cells (VSELs) (Kucia et al., 2006; Kucia et al., 2007). The existence of these cells has been confirmed by at least 50 independent research groups (Supplementary Table 1). VSELs are quiescent cells that become activated during stressful situations. As demonstrated, they are mobilized into the peripheral blood (PB) in response to stress and tissue or organ damage (Ratajczak et al., 2011a; Wojakowski et al., 2009; Marlicz et al., 2012; Drukala et al., 2012; Kucia et al., 2008). Their numbers decline with age (Ratajczak et al., 2010). The presence of these cells in postnatal tissues, which may differentiate across germ layers, challenges the established stem cell hierarchy within the adult stem cell compartment. Therefore, the discovery of VSELs provides new insights into tissue and organ rejuvenation, aging, longevity, the adult stem cell hierarchy, and the origin of malignancies.

### Developmental origin of VSELs and their potential link to blastocyst inner cells mass PSCs, epiblast cells, and migrating primordial germ cells (PGCs)

**FIGURE 2:**
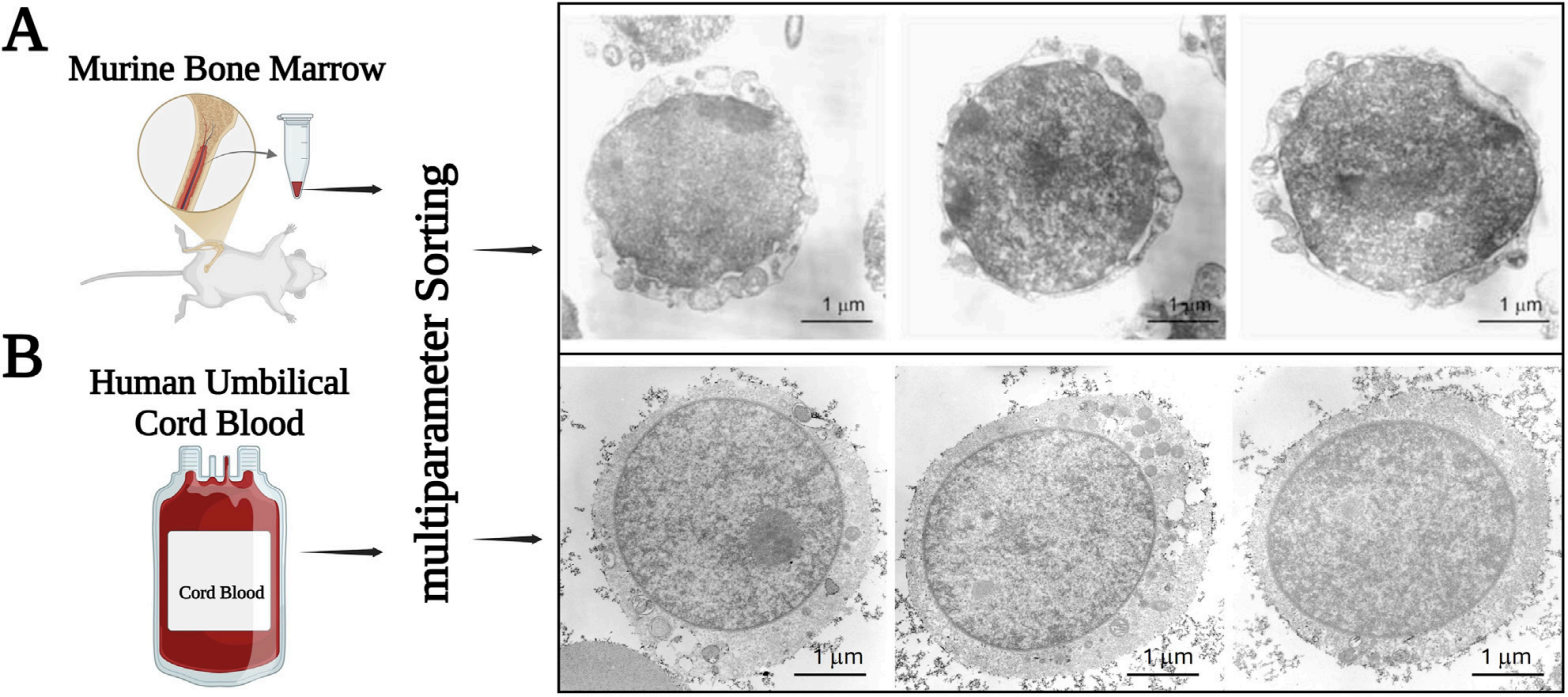
Transmission electron microscopy (TEM) pictures of murine bone marrow- and human umbilical cord blood-purified VSELs. Panel **(A)** murine VSELs are small, possess a relatively large nucleus surrounded by a narrow rim of cytoplasm. At the ultrastructural level the narrow rim of cytoplasm possesses a few mitochondria, scattered ribosomes, small profiles of endoplasmatic reticulum and a few vesicles. The nucleus is contained within a nuclear envelope with nuclear pores. Chromatin is loosely packed and consists of euchromatin. Panel **(B)** human VSELs are small and similarly as murine VSELs possess a relatively large nucleus surrounded by a narrow rim of cytoplasm. At the ultrastructural level, this narrow rim of cytoplasm possesses a few round mitochondria, scattered ribosomes, small profiles of endoplasmatic reticulum and a few vesicles. The nucleus is contained within a nuclear envelope with nuclear pores. Chromatin is loosely packed and consists of euchromatin. These pictures are adopted from published papers (references # 24 and # 25) after obtaining permission from the Leukemia Journal.

We envision VSELs being deposited in developing organs during embryogenesis, serving as a backup population for monopotent tissue-committed stem cells (Figure 1B). Our single-cell cDNA libraries revealed that these cells, purified by multiparameter FACS from murine or human hematopoietic tissues, consist of a population of small Sca-1^+^lin^−^CD45^−^ cells in mice and either CD133^+^lin^−^CD45^−^ or CD34^+^lin^−^CD45^−^ cells in humans, demonstrating some heterogeneity in the expression of specific early developmental and lineage-specific genes (Shin et al., 2012). Our molecular analysis of gene expression in murine and human VSELs supports the hypothesis that these cells originate from precursors related to PSCs found in the inner cell mass (ICM) of the blastocyst, as well as the epiblast and germline. To further this notion, VSELs express several genes characteristic of PSCs (such as *Oct-4, SSEA, Nanog, Sox-2, Klf4, and Rex-1*), epiblast stem cells (EpiSCs) (including *Gbx2, Fgf5,* and *Nodal*), and migrating primordial germ cells (PGCs) (like *Stella, Prdm14, Fragilis, Blimp1, Dppa2, Dppa4, Mvh, Nanos3,* and *Dnd1*) (Shin et al., 2012; Ratajczak et al., 2017). However, VSELs are more differentiated than blastocyst ICM-derived PSCs and share several markers with the more differentiated EpiSCs (Shin et al., 2012; Ratajczak et al., 2017). The expression of some of these genes detected in bulk preparations of mRNA isolated from these cells was subsequently confirmed through immunostaining. The detailed transmission electron microscopy pictures of murine and human VSELs shown in Figure 2 were published in our earlier papers (Kucia et al., 2006; Kucia et al., 2007). We also recently published more detailed gene expression data from single-cell seqRNA analysis (Jarczak et al., 2024).

In detail, the relationship between VSELs, germline, and PGCs is evidenced by the expression of several functional pituitary and gonadal sex hormone receptors in VSELs (Mierzejewska et al., 2015; Abdelbaset-Ismail et al., 2016). From an evolutionary perspective, the germline is considered immortal as it transfers DNA and mitochondria to the next-generation. Living organisms, along with their various stem cell compartments, develop from the fusion of gametes derived from PGCs. VSELs express multiple markers characteristic of PGCs, supporting the notion that the most primitive stem cells found in adult tissues are related to PGCs. Additionally, chromatin immunoprecipitation (ChIP) results in VSELs showed that the promoter of the PGCs characteristic gene, *Stella,* exhibits transcriptionally active histone modifications, including acetylated histone 3 [H3Ac] and trimethylated lysine four of histone 3 [H3K4me3]. In contrast, it is less enriched for transcriptionally repressive histone markers such as dimethylated lysine nine of histone 3 [H3K9me2] and trimethylated lysine 27 of histone 3 [H3K27me3] (Shin et al., 2012; Ratajczak et al., 2010).

VSELs also show bivalent domains at the promoters of homeodomain-containing transcription factors (TFs), including *Sox21, Nkx2.2, Dlx1, Lbx1h, Hlxb9, Pax5,* and *HoxA3* (Shin et al., 2012). Bivalent domains are typical of PSCs and indicate a chromatin structure state where transcriptionally opposing histone codes coexist within the same promoter of homeodomain-containing transcription factors (Voigt et al., 2013).

**FIGURE 3:**
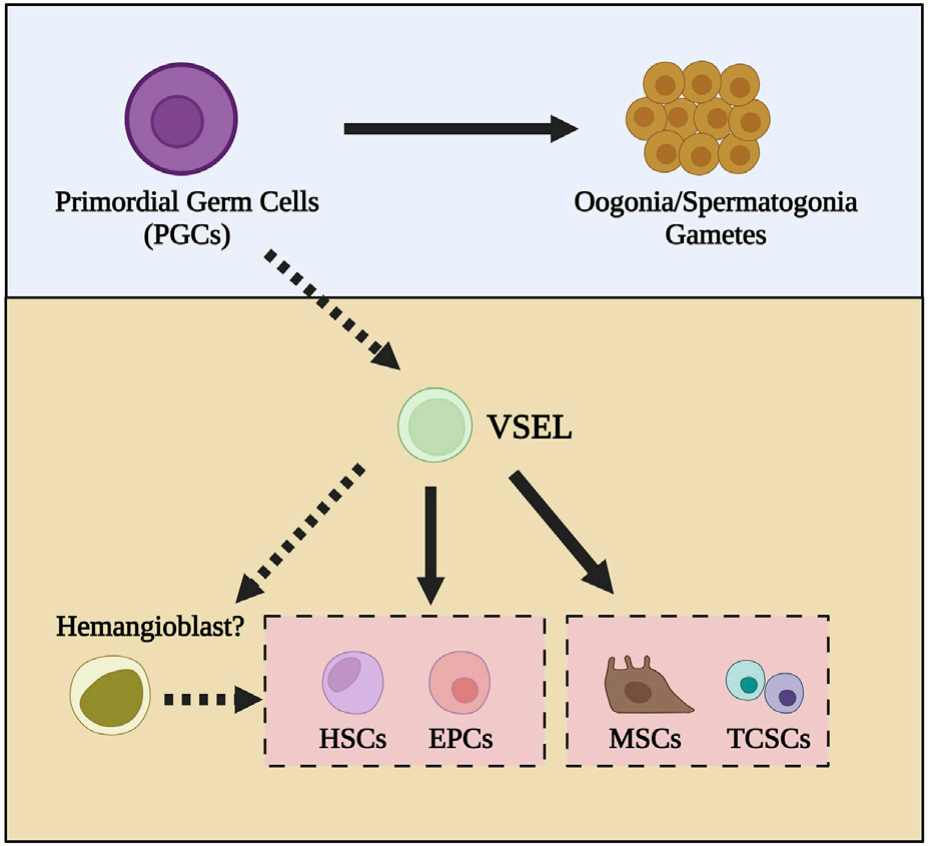
The developmental interrelationship among primordial germ cells (PGCs), very small embryonic-like cells (VSELs), hematopoietic stem cells (HSCs), mesenchymal stem cells (MSCs), endothelial progenitor cells (EPCs), and tissue-committed stem cells (TCSCs). We propose that VSELs share several similarities with migrating PGCs. They may serve as precursors to various types of adult stem cells, including bone marrow-derived tissue-committed stem cells (TCSCs), mesenchymal stem cells (MSCs), hematopoietic stem cells (HSCs), and endothelial progenitor cells (EPCs). However, as shown, the differentiation of VSELs into HSCs and EPCs, which involves a hemangioblast intermediate, requires further investigation.

We envision that, as depicted in Figure 3, VSELs could be a derivative of migrating late PGCs and give rise to HSCs, EPCs, and MSCs in BM. In other tissues, they serve as backup for monopotent tissue-committed stem cells.

### VSELs are purified from adult tissues by multiparameter FACS sorting

It is widely accepted that stem cells have a distinct morphology (i.e., small size and a lymphocyte-like appearance), express a specific set of surface markers (i.e., CD133^+^, CD34^+^, Lin^–^), show low accumulation of certain metabolic fluorochromes (e.g., Rhodamine 123, Pyronin Y, or Hoe3342), and exhibit variations in the activity of specific enzymes (e.g., aldehyde dehydrogenase [ALDH]). All of these characteristics are important for SC identification and purification strategies using a FACS sorter (Zuba-Surma and Ratajczak, 2010).

Therefore, VSELs can be easily isolated from adult tissues, including bone marrow (BM), umbilical cord blood (UCB), or mobilized peripheral blood (mPB), using multiparameter cell sorting that utilizes the Sca-1 antigen in mice and CD34 or CD133 in humans as positive markers (Kucia et al., 2006; Kucia et al., 2007; Zuba-Surma and Ratajczak, 2010). Furthermore, they are lineage-negative and do not express the hematopoietic CD45 marker. Several groups that accurately followed the original protocol for their isolation published by us in *Current Protocols of Cytometry,* or contacted us for assistance have successfully identified these small cells in postnatal tissues (Zuba-Surma and Ratajczak, 2010).

Since we used two alternative antigens to purify CD34^+^ or CD133^+^ VSELs, we recently compared both populations of these cells, purified as CD133^+^lin^−^CD45^−^ or CD34^+^lin^−^CD45^−^ VSELs. We noticed that CD133^+^lin^−^CD45^−^ cells are rarer and express mRNA for pluripotency markers Oct-4 and Nanog at higher levels, as well as the stromal-derived factor-1 (SDF-1) CXCR4 receptor, and extracellular ATP purinergic receptors from P2X1-P2X7 family which regulate the trafficking of these cells (Ratajczak et al., 2004; Bujko et al., 2024). However, neither cell population showed significant differences in the expression of proteins assigned to primary biological processes according to the UniProt database (http://geneontology.org/). Next, through analyzing cellular metabolic processes, we observed an increase in the expression of proteins involved in nitrogen metabolism and cyclic compounds, heterocycle metabolism, nucleobase-containing compound metabolism, nucleic acid processing, RNA metabolism, DNA regulation, and negative regulation of protein metabolism in CD133+ VSELs (Jarczak et al., 2024). Simultaneously, the proteome analysis of CD34^+^ VSELs revealed an increase in cellular responses related to nitrogen compounds and responses to cytokines, irradiation, chemical stress, reactive oxygen species, and oxidative stress exposure. This indicates that despite having similar morphology, biological effects, and specification features, CD133^+^lin^−^CD45^−^ VSELs differ slightly from their CD34^+^lin^−^CD45^−^ counterparts; these differences were explored in more detail through single cell seqRNA analysis, which will be described later (Jarczak et al., 2024).

It is important to note that Dr. Bhartyja’s group successfully isolates VSELs from hematopoietic tissues using differential centrifugation of cell suspension (Bhartiya, 2017; Parte et al., 2011; Bhartiya et al., 2012; Anand et al., 2013; Bhartiya et al., 2023).

### The consequences of epigenetic modification of parentally imprinted genes explain the quiescent state of VSELs

Although murine VSELs exhibit several features of PSCs, they are quiescent and do not fulfill two “gold standard” *in vivo* criteria of pluripotency expected from PSCs: i) they do not complete blastocyst development, and ii) they do not form teratomas after transplantation into immunodeficient mice (Ratajczak et al., 2019; Bhartiya et al., 2012; Ratajczak et al., 2011b; Ratajczak et al., 2011c). These *in vivo* pluripotency criteria are characteristic of ESCs and iPSCs; however, they do not apply to PGCs, which, despite being stem cells endowed with developmental totipotency, do not comply with this definition (Damdimopoulou et al., 2016; Shi et al., 2017; Surani et al., 2007). To explain these differences, PGCs modify the methylation of specific parentally imprinted genes, and this modification (the erasure of imprinting) hinders them from proliferating, completing blastocyst development, and forming teratomas (Maatouk et al., 2006; Sriraman et al., 2015; Surani et al., 2008; Bartolomei and Ferguson-Smith, 2011; Hajkova et al., 2002). Given that VSELs, as we envision them, share several molecular characteristics with PGCs, we investigated whether a similar mechanism is responsible for the quiescent state of VSELs (Ratajczak et al., 2011d; Ratajczak M. Z. et al., 2013; Maj et al., 2015).

**FIGURE 4:**
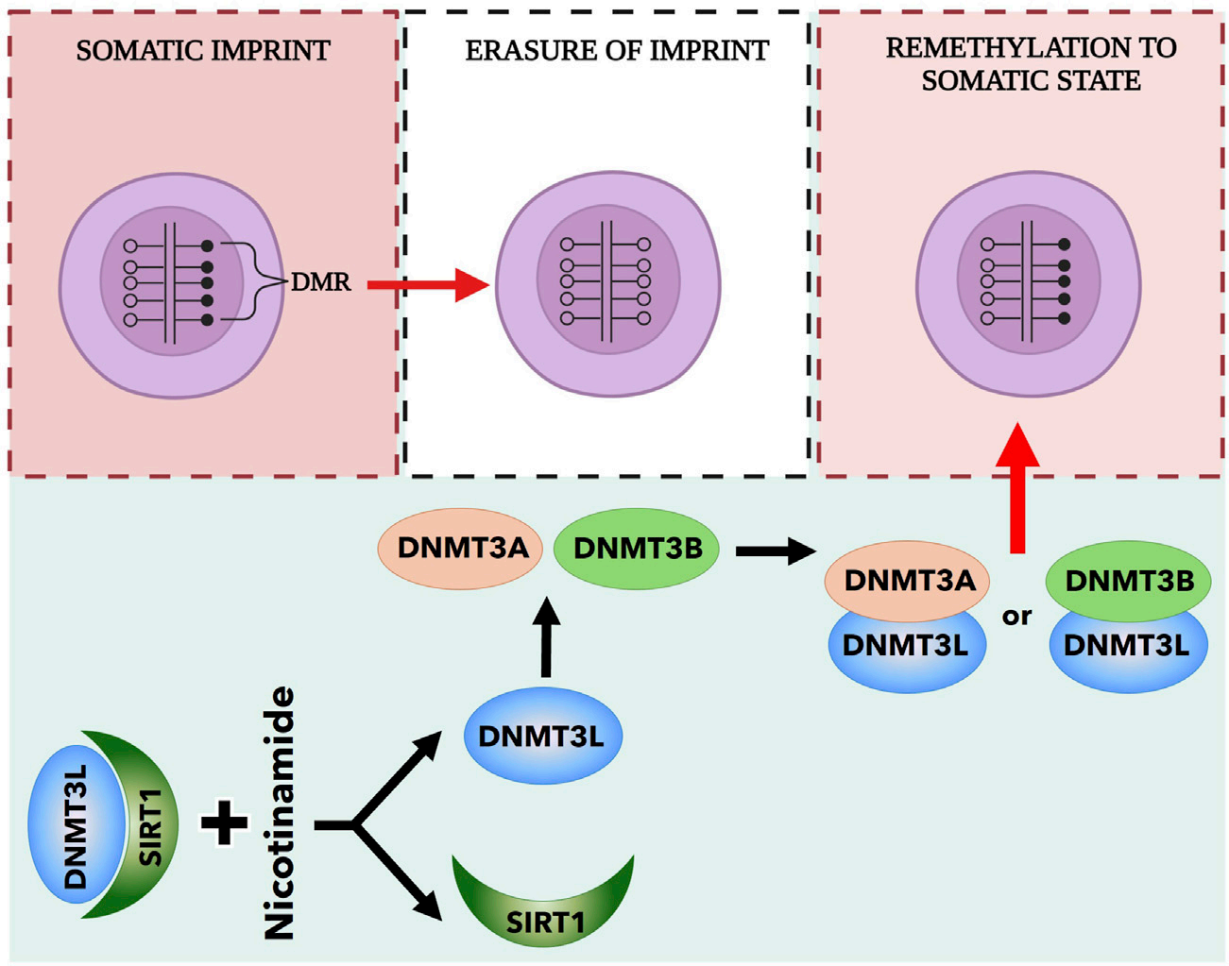
Reinstate the somatic imprint in the differently methylated region (DMR) of the tandem gene Igf-2-H19. Very Small Embryonic Like Stem cells (VSELs) erase paternal imprints on the Igf2-H19 DMR. This action prevents their proliferation and keeps them in a quiescent state. *De novo* methyltransferases can then remethylate this erased imprint. This process requires the coordinated actions of DNMT3A and DNMT3B, along with DNMT3L. DNMT3L receives support from histone deacetylase (sirtuin). When nicotinamide is introduced, DNMT3L is released from sirtuin and may remethylate the erased locus. We utilize this nicotinamide effect in the *ex vivo* expansion of VSELs.

We noticed that murine BM-derived VSELs erase the paternally methylated imprints at the *Igf2–H19* and *Rasgrf1* loci; however, they simultaneously hypermethylate the maternally methylated imprints at the locus encoding the Igf2 receptor [*Igf2R*]*, as well as at Kcnq1-p57* ^*KIP2*^ and *Peg1* (Shin et al., 2009; Ratajczak et al., 2011d; Ratajczak M. Z. et al., 2013; Maj et al., 2015; Heo et al., 2017). Notably, the pattern of parental imprinting erasure in VSELs differs slightly from that in PGCs, primarily affecting paternally imprinted genes such as *Igf2-H19 and Rasgrf1*. As a result of the erasure of the differently methylated region (DMR) at the *Igf2-H19* tandem gene, non-coding mRNA H19 is transcribed. This produces miRNA species that inhibit Igf1 receptor (Igf1R) expression while simultaneously downregulating mRNA expression for IGF-2. Additionally, VSELs express Igf2R, a negative regulator of systemic IGF-2 levels that promotes its degradation after binding. Furthermore, the erasure of the DMR at the *Rasgrf1* gene impacts Ras-Grf1 signaling downstream of the insulin receptor (InsR) and Igf1R, rendering these cells resistant to Insulin/IGF-1 signaling. These changes in the expression of parentally imprinted genes, primarily affecting insulin-like growth factors/insulin signaling, protect VSELs from premature proliferation and specification into tissue-committed stem cells. This somehow ultimately prevents their premature depletion from adult tissues. Our strategy to expand *ex vivo* quiescent VSELs is based on reinstating proper methylation in the DMRs of these imprinted genes to a somatic status, as illustrated in Figure 4 and described below.

We observed that *de novo* DNA methyltransferase 3 L (DnmT3L), which is crucial for the methylation of regulatory regions of paternally imprinted genes, is chaperoned in stem cells by histone deacetylase sirtuin-1 (Sirt-1). To free DnmT3L from the inhibitory effects of Sirt-1, we used nicotinamide and valproic acid, both of which act as Sirt-1 inhibitors (Figure 4). With the addition of nicotinamide and valproic acid, we were able to revert the quiescent state of very small embryonic-like stem cells (VSELs), which began in a specialized expansion medium, to proliferate and expand *ex vivo* (Ratajczak et al., 2017; Heo et al., 2017). Importantly, this proliferation did not pose a risk of teratoma formation from these cells. In our experiments, VSELs expanded up to 3000-fold in Dulbecco’s medium supplemented with artificial “knock-out” serum in the presence of nicotinamide or valproic acid (Ratajczak et al., 2017; Heo et al., 2017). We enhance this process by using a cocktail of follicle-stimulating hormone (FSH), luteinizing hormone (LH), bone morphogenetic protein-4 (BMP-4), and kit ligand (KL). The expanded *ex vivo* cells consist of small VSEL-like cells and larger, more differentiated derivatives. This strategy has been patented, and we are currently focused on optimizing the expansion protocol for both murine and human VSELs for testing in non-hematopoietic tissue/organ injury models. Recently, another group successfully employed a pyrimidoindole derivative (UM171) (Fares et al., 2014) for efficient *in vitro* expansion of VSELs (Lahlil et al., 2018).

In conclusion, VSELs highly express growth-repressive genes (*H19, p57* ^*KIP2*^ *,* and *Igf2R*) while downregulating growth-promoting genes (*Igf2* and *Rasgrf1*) (Shin et al., 2009; Ratajczak et al., 2011d; Ratajczak M. Z. et al., 2013; Maj et al., 2015; Heo et al., 2017). These epigenetic changes induce a growth-repressive state in VSELs and contribute to their quiescent state primarily by diminishing proteins involved in insulin-like growth factors 1 and 2 (IGF-1 and IGF-2) and insulin signaling, including those encoded by the *Igf2–H19* and *Rasgrf1* loci. Based on these findings, it became clear that the re-methylation of erased differently methylated regions (DMRs) at paternally imprinted loci by *de novo* DNA methyltransferases (DnmTs) would reverse the quiescent state of VSELs (Ratajczak et al., 2017). This strategy was employed to expand these cells *ex vivo*.

### The tissue specification of VSELs reveals their pluripotent/multipotent character

Several elegant studies have demonstrated VSEL specification across germ layers, showing that injected purified VSELs contribute to hematopoiesis (Ratajczak et al., 2011b; Ratajczak et al., 2011c), osteogenesis (Havens et al., 2013; Havens et al., 2014), angiogenesis (Guerin et al., 2015; Domingues et al., 2022), as well as to the myocardium (Dawn et al., 2008; Zuba-Surma et al., 2011), liver (Chen et al., 2015), and pulmonary alveolar epithelium (Kassmer et al., 2013; Kassmer et al., 2012). The well-established presence of chimerism in the organs targeted by VSEL therapy indicates the potential of these cells to differentiate into various tissues. As postulated, VSELs occupy the pinnacle of the stem cell hierarchy in normal bone marrow, giving rise to hematopoietic stem cells (HSCs), mesenchymal stem cells (MSCs), and endothelial progenitor cells (EPCs).

VSELs are at the top of the hematopoietic/lymphopoietic lineage. Based on the migratory path of PGCs and their intersection with the developing sites of hematopoiesis—initially at the base of the yolk sac and later in the aorta-gonad-mesonephros region (AGM)—we hypothesized that VSELs could function as precursors to HSCs, related to PGCs (Kritzenberger and Wrobel, 2004; Ohtaka et al., 1999; Rich, 1995; Ratajczak, 2015). Our initial experiments, which involved the hematopoietic specification of VSELs on OP-9 stromal cells used for the hematopoietic specification of ESCs, supported this hypothesis (Ratajczak et al., 2011b; Ratajczak et al., 2011c). Recently, we have utilized an artificial serum medium enriched with DNA modifiers such as nicotinamide or valproic acid to encourage the re-methylation of erased DMRs in paternally imprinted genes, along with a mix of selected factors, including the aforementioned sex hormones (FSH, LH, danazol) and BMP-4 and KL, to expand VSELs and induce hematopoietic specification. We observed that highly quiescent VSELs in murine BM entered the cell cycle and increased in number, as confirmed by bromodeoxyuridine (BrdU) accumulation (Mierzejewska et al., 2015). This responsiveness of VSELs to sex hormones further reinforces a developmental link between these cells and PGCs (Kritzenberger and Wrobel, 2004; Ohtaka et al., 1999; Rich, 1995; Ratajczak, 2015; Scaldaferri et al., 2015).

VSELs are at the top of the hierarchy of mesenchymal stem cells (MSCs). Taichman et al. demonstrated that murine BM-sorted VSELs possess the essential characteristics of precursors for MSCs, as they can differentiate *in vivo* into various mesenchymal lineages and generate osseous tissues at low density (Havens et al., 2013; Havens et al., 2014). Furthermore, VSELs derived from GFP + mice formed bone-like structures when implanted into SCID mice. To confirm that this represents true bone formation activity dependent on VSELs, stromal cells were harvested from Col2.3∆TK mice and implanted into SCID mice to create thymidine kinase-sensitive ossicles. After 1.5 months, these ossicles were subsequently injected with 2000 GFP + murine VSELs (Havens et al., 2013; Havens et al., 2014). As evidence, the authors demonstrated the colocalization of GFP-expressing cells with the osteoblast-specific marker Runx-2, the endothelial marker CD31, and the adipocyte marker PPAR. Therefore, VSEL-derived MSCs exhibit differentiation into three mesenchymal lineages. A similar bone-forming potential of human VSELs has also been demonstrated *in vivo* in an immunodeficient mouse model (Havens et al., 2013; Havens et al., 2014).

VSELs as a source of endothelial progenitor cells. Evidence has accumulated that VSELs can differentiate into endothelial progenitor cells. As reported by Smadja et al., BM-residing VSELs are mobilized into the PB of patients with critical limb ischemia. Purified BM-VSELs cultured in angiogenic media acquired a mesenchymal phenotype based on the expression of CD90^+^ and the positivity of the Thy-1 gene (Guerin et al., 2015). More importantly, VSELs facilitated post-ischemic revascularization in immunodeficient mice and attained an endothelial phenotype either *in vitro* when cultured with VEGF-B (Cdh-5 gene positivity) or *in vivo* in Matrigel implants (human CD31 positive staining in neo-vessels from plug sections) (Guerin et al., 2015). Supporting these exciting results, we observed a high level of *Flk2* transcript expression in highly purified VSELs. Recently, in our collaborative paper with Dr. Smadja’s group, we demonstrated for the first time that human BM-purified VSELs differentiate into true vasculogenic progenitor endothelial cells in chemically defined media supplemented with UM-171, nicotinamide, FGF-2, VEGF-A, and BMP-4 (Domingues et al., 2022). VSEL-derived endothelial progenitors exhibited the same endothelial morphology, phenotype, and secretory potential as UCB endothelial progenitors and could form functional vessels in a Matrigel implant model (Domingues et al., 2022). In conclusion, VSELs represent a promising new source of therapeutic cells that may give rise to human endothelial lineage cells.

VSELs are potential precursors of cardiomyocytes. It has been reported that freshly purified BM-derived VSELs from GFP^+^ mice, when injected into the hearts of mice following ischemia/reperfusion injury, improved both global and regional left ventricular (LV) systolic function and reduced myocyte hypertrophy in the surviving tissue (as shown by histology and echocardiography) compared to vehicle-treated controls (Dawn et al., 2008; Zuba-Surma et al., 2011). Importantly, some newly formed GFP^+^ cardiomyocytes and capillaries were detected in the infarcted myocardium. This data suggests that VSELs could be promising candidates for cardiac regeneration. We plan to repeat these experiments with *ex vivo* expanded VSELs and anticipate a more substantial regeneration of the myocardium after injecting a higher number of these cells. In another study by Yuzhen et al., rat BM VSELs-derived embryoid-like bodies cultured in soft agarose in the presence of leukemia inhibitory factor and FGF-2 differentiated into cardiomyocytes and endothelial cells (Wu et al., 2012). Notably, these cells derived from male rat embryoid bodies reduced the scar area and significantly improved cardiac function in a female rat following ischemia/reperfusion injury. In this model, the authors convincingly demonstrated the presence of the Y chromosome in male VSELs-derived cardiomyocytes and endothelial cells in transplanted female mice (Wu et al., 2012).

A recent report examined human heart tissue collected from healthy subjects and those with ischemic heart disease for the presence of VSELs based on the CD45-/CD133+/SSEA4+ phenotype. The number of these cells in epicardial and endocardial tissues was compared across various age groups. Human VSELs are present in adult hearts, with a decreasing prevalence as age increases (El-Helw et al., 2020).

VSELs and liver regeneration. Zhou et al. reported that VSELs can differentiate into hepatic cells. According to the study, murine VSELs exposed to hepatocyte growth factor and kit ligand significantly reduced serum ALT and AST levels when transplanted into mice with CCl4-induced liver injury (Chen et al., 2015). Consequently, it was concluded that VSELs play a role in repairing liver injury. Furthermore, these intriguing findings need to be replicated using *ex vivo* expanded VSELs specialized into the hepatocyte lineage.

VSELs and their contribution to the lung alveolar epithelium. As previously mentioned, ELH stem cells isolated from murine bone marrow share several characteristics with VSELs. Krause et al. investigated whether VSELs purified from bone marrow align with ELH stem cells in a model of lung alveolar epithelium regeneration. In this elegant study, mice with irradiation-induced lung injury were injected with i) bone marrow-purified VSELs, ii) hematopoietic stem/progenitor cells, and iii) other bone marrow-derived non-hematopoietic cells (Kassmer et al., 2013; Kassmer et al., 2012). VSELs exhibited the highest rate of lung epithelial cell formation, as reported (Kassmer et al., 2013; Ciechanowicz et al., 2021). To gather more evidence, VSELs were purified from donor mice expressing H2B–GFP under a type 2 pneumocyte-specific promoter and were transplanted into mice with lung injury. These cells differentiated into type 2 pneumocytes (Kassmer et al., 2012). This process also ruled out the likelihood of a fusion phenomenon. In a follow-up experiment, it was demonstrated that bone marrow-derived VSELs engraft as lung epithelial progenitor cells following bleomycin-induced lung injury. Using a reporter mouse with the H2B-GFP fusion protein driven by the murine surfactant protein C (SPC) promoter, we tested whether bone marrow-purified VSELs could differentiate into AT2 and BASC cells, functioning as progenitor cells in the lung epithelium in mice with lung damage caused by bleomycin exposure. As reported, 21 days after their injection into the mice, VSELs, but not non-VSEL/non-HSCs, differentiated into phenotypic AT2 and BASC cells, consistent with previous data in irradiated recipients (Kassmer et al., 2013; Ciechanowicz et al., 2021). This was subsequently confirmed *in vitro* through organoid assays, indicating that VSEL-derived AT2 and BASC maintained their physiological potential for differentiation and self-renewal (Kassmer et al., 2013; Ciechanowicz et al., 2021).

VSELs and their specification into gametes. VSELs were initially identified in hematopoietic tissues, but their presence in murine and human gonads was discovered shortly thereafter. Two independent leading research groups, those of Dr. Bhartiya (Bhartiya, 2017; Parte et al., 2011; Bhartiya et al., 2012; Anand et al., 2013) and Dr. Viran-Klunt (Virant-Klun et al., 2008; Virant-Klun et al., 2009; Virant-Klun, 2018), identified very small cells in human and murine ovaries and later in testes that resemble VSELs. Both groups proposed that these cells could serve as an alternative source of oocytes and sperm for patients with damaged gonads due to high-dose chemotherapy or for women with primary ovarian failure. Support for this has been provided by compelling evidence that VSELs can be isolated from the ovarian surface epithelium of both young and postmenopausal women, as well as from seminiferous tubules in the testes, where they can differentiate into gamete-like cells (Virant-Klun et al., 2008; Virant-Klun et al., 2009; Virant-Klun, 2018). Interestingly, they also established spermatogenesis in the testes after high-dose chemotherapy, which was followed by the injection of MSCs or Sertoli cells (Bhartiya et al., 2012; Anand et al., 2013). Recently, it was reported that VSELs isolated from the ovary differentiate into oocyte-like cells and release the zona pellucida in response to sperm cells, marking the first step in the fertilization process (Virant-Klun, 2018). These exciting findings open up new possibilities for reproductive medicine.

## The role of VSELs in aging

Healthy aging is a key goal of regenerative medicine, and the proper functioning of stem cells in tissue rejuvenation and regeneration is vital. These cells replace those that become depleted over time. The processes of cell replacement differ among various organs. VSELs, which serve as a source of tissue-committed monopotent stem cells, are significant. The number of VSELs declines in tissues and organs as one ages, and it has been suggested that prolonged insulin and insulin-like growth factor signaling may diminish the pool of these cells. We conclude that over their lifespan, these cells gradually diminish in response to stress and are “consumed in a fire of insulin and insulin-like growth factor signaling” under both normal and stressful conditions.

Research has shown that the number of VSELs correlates with longevity in certain long-lived murine strains (Shin et al., 2009; Sluczanowska-Glabowska et al., 2012; Kucia et al., 2011). This correlation is especially evident in animals like Laron and Ames dwarf mice, which exhibit undetectable insulin-like growth factor - one levels in their peripheral blood (Shin et al., 2009; Sluczanowska-Glabowska et al., 2012; Kucia et al., 2011; Kucia et al., 2013; Ratajczak et al., 2011e). These mice display an increased count of VSELs in their bone marrow. Interestingly, they also possess more VSELs in their ovaries and experience extended lifespans alongside prolonged reproductive functions. We successfully bred 2.5-year-old Laron dwarf female mice, even though the reproductive period typically concludes by 1.5 years in their normal counterparts (Grymula et al., 2014). This data further supports the potential role of VSELs as precursor cells for gametes.

Normal animals exposed to high levels of insulin and insulin-like growth factor −1 signaling experience premature aging and deplete their pool of VSELs (Ratajczak et al., 2010). In contrast, their numbers can increase in experimental animals through caloric restriction (Grymula et al., 2014), regular exercise (Marycz et al., 2016), and DNA modifiers like nicotinamide or valproic acid (Ratajczak et al., 2017).

### The role of VSELs in responding to tissue and organ injuries

VSELs can be mobilized into peripheral blood in response to various tissue and organ injuries, as shown in mice and humans suffering from heart infarction (Wojakowski et al., 2009), stroke (Paczkowska et al., 2009), skin burns (Drukala et al., 2012), and intestinal inflammation (Marlicz et al., 2012). Their numbers also increase in the peripheral blood of cancer patients (Bhartiya et al., 2023). VSELs migrate during development, initially in the embryo alongside PGCs, and later settle in developing tissues. Consequently, they are migratory cells of early development. We envision that despite circulating in peripheral blood at low numbers, they robustly respond to chemotactic factors released at sites of tissue injury, such as SDF-1, S1P, eATP, and HGF (Ratajczak et al., 2004; Bujko et al., 2024).

The responsiveness of VSELs in stressful situations and their mobilization into peripheral blood (PB) is linked to the expression of receptors for chemotactic factors that regulate stem cell trafficking. This includes the CXCR4 receptor for stroma-derived factor-1 (SDF-1), the sphingosine-1 phosphate receptor, and receptors from the purinergic signaling P2X family, which are activated by extracellular ATP (Ratajczak et al., 2004; Bujko et al., 2024). Although VSELs are mobilized into PB in response to stress and various pathologies, their numbers are often too low to significantly repair tissue or organ damage. However, since we observe an increase in the number of these cells in PB after several types of solid organ or tissue injuries, as well as after strenuous exercise, they may aid in the regeneration of minor injuries, such as, for example, hypoxia-mediated muscle damage in marathon runners (Marycz et al., 2016).

### The potential role of VSELs in cancer

Whether cancer originates in differentiated somatic cells or the stem/progenitor cell compartment remains a matter of debate (Tu et al., 2023; Ratajczak et al., 2018; Jassim et al., 2023). Recent evidence indicates that tumors arise from the accumulation of mutations and the maturation arrest of normal stem/progenitor cells, rather than from the dedifferentiation of already differentiated cells. The morphology of several tumors resembles developmentally early tissues. Furthermore, carcinogenesis often responds to chronic irritation, inflammation, and tissue damage, likely proceeding by misappropriating the homeostatic mechanisms governing tissue repair and stem cell self-renewal. The increase in cancer incidence linked to chronic injury strongly supports the view of cancer as a continuous state of repair, implying a role for stem cells in this process. Interestingly, tumors frequently express early developmental markers characteristic of the germ line lineage (Ratajczak et al., 2009). This raises a significant question: Do these common features of tumors reflect the dedifferentiation of mutated cells from the somatic lineage where cancer develops, or do they emerge because cancer originates in the most primitive stem cells closely related to the germ line? The identification of primitive germ line-derived VSELs has introduced the possibility that cancer may arise from these cells. This concept may support the over 100-year-old theories of embryonic rest or germ line origin hypotheses of cancer development proposed in the 19th century by Virchow and Connheim (Virchow, 1855). VSELs express several cancer-testis antigens found in various types of malignancies. Notably, Dr. Bhartyja’s group reported an increase in circulating PB VSELs in several human malignancies (Bhartiya et al., 2023). If these cells are mutated, they could be the source of some malignancies, or their increased circulation might indicate their detection of developing tumors as damaged tissues. Unfortunately, they are likely to provide stroma and vasculature that promote the development of malignancies. Nevertheless, further studies are necessary to determine the potential role of VSELs in tumorigenesis.

### VSEL characterization in the era of OMIC strategies

Our molecular analysis of VSELs, as described earlier, focuses on examining bulk mRNA isolated from highly purified VSELs. We can more effectively characterize these cells at the single-cell level by utilizing single-cell RNA sequencing (scRNA-seq).

#### Single-cell seqRNA–A new era in molecular characterization of VSELs

To investigate the composition of gene expression and the diversity of human UCB CD133^+^ and CD34^+^ VSELs, and to reassess the data obtained from bulk mRNA analysis as described earlier, we conducted scRNA-seq (10 × Genomics, United States) using cells purified with the MoFlo Astrios EQ Cell Sorter. A multi-step procedure for preparing single-cell libraries enabled us to capture 3,706 CD133^+^lin^−^CD45^−^and 1,592 CD34^+^lin^−^CD45^−^ VSELs, which were then identified based on differential gene expression and categorized into specific cell subpopulations (clusters) (Jarczak et al., 2024).

The groundbreaking observation from our single-cell RNA sequencing analysis indicated that both human VSEL populations purified from UCB express similar molecular signatures to those reported for murine BM-derived VSELs, forming a heterogeneous group of small cells. These quiescent small cells may respond to various stimuli in developing and postnatal organisms, potentially becoming primed or specified across germ layers into different types of stem cells.

We previously reported that murine BM-purified VSELs expressed (i) a transcriptome resembling that of murine ESCs, (ii) key cell cycle checkpoint genes, (iii) low levels of genes involved in protein turnover and mitogenic pathways, and (iv) high levels of the enhancer of zeste *drosophila* homolog 2 (Ezh2) (Shin et al., 2009). In a recently published scRNA-seq study, we observed a similar gene expression pattern in human CD34^+^ and CD133^+^ UCB-purified VSELs (Jarczak et al., 2024). This confirms the developmental homology between these cells isolated from small and large mammals. Furthermore, unsupervised clustering revealed subpopulations of UCB-purified VSELs that express genes (i) annotated to germline compartments, (ii) regulated by parental imprinting, (iii) responding to early developmental fate decisions, (iv) encoding transcription factors involved in differentiation and development, including the homeobox gene family, (v) expressing innate immunity and purinergic signaling genes, and (vi) involved in hematopoietic specification (Kucia et al., 2006). However, when analyzing this data, we must remember that BM-derived VSELs mobilized during delivery to UCB may be preferentially specified by a BM microenvironment into hemato/lymphopoietic cells, mesenchymal stem cells, and endothelial progenitors, while also expressing some mRNA species from embryonic development. We are currently conducting proteomic and metabolic analyses on highly purified human VSELs.

## Conclusions and future directions

**FIGURE 5:**
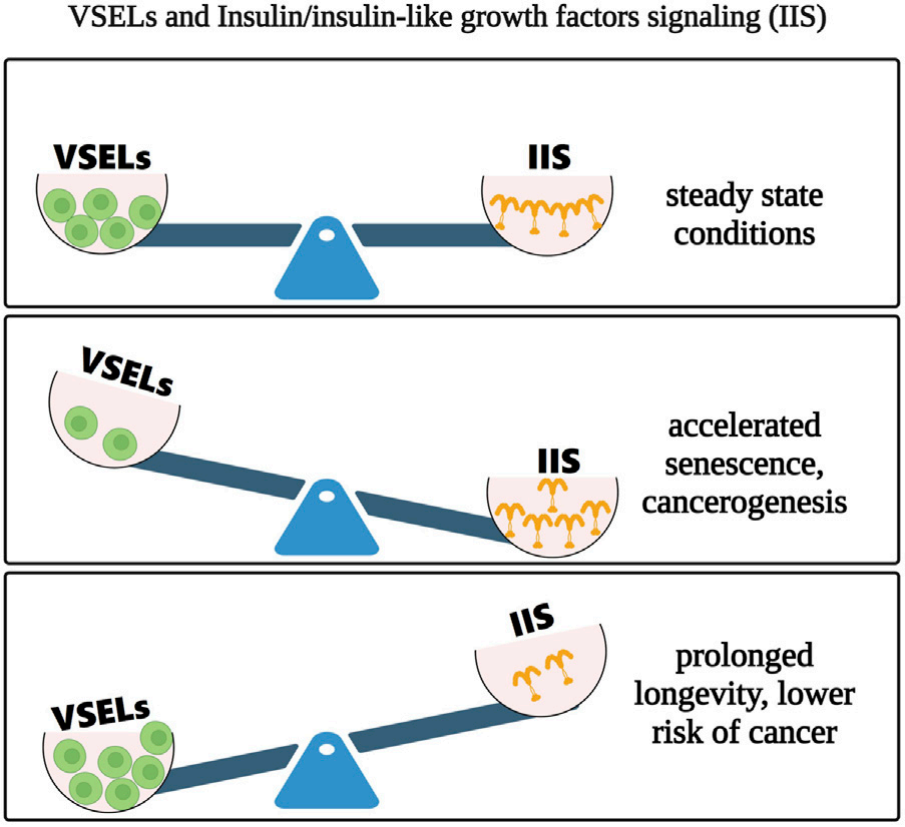
Changes in the balance between VSEL numbers and insulin/insulin-like growth factor signaling (Upper panel) Proper insulin/insulin-like growth factor signaling maintains the necessary number of VSELs in adult tissues (Middle panel) Increased and prolonged insulin/insulin-like growth factor signaling activates and prematurely depletes VSELs from the tissues, resulting in accelerated aging and a potential induction of cancer (Lower panel) In contrast, balanced low insulin/insulin-like growth factor signaling (e.g., caloric restriction, regular physical activity, metformin medication) preserves the essential number of VSELs in a quiescent state within adult tissues.

VSELs, with their broad differentiation potential, have emerged as a new candidate population of cells for clinical applications. This is now feasible due to our development of a model for *ex vivo* expansion of these rare cells. Importantly, there is no evidence that these cells, isolated from adult tissues, can form teratomas. We propose that pluripotent VSELs be studied further, as they may offer solutions to several challenges posed by ESCs and iPSCs in regenerative medicine. However, since these cells could be prematurely depleted from adult tissues in response to increased insulin/insulin-like growth factor signaling, it is challenging for modern pharmacology to create more specific medications for regulating proper insulin signaling. Alongside caloric restriction and regular exercise, some positive effects of metformin on VSEL numbers and longevity further underscore the necessity of developing more effective and targeted drugs aimed at insulin/insulin-like growth factor signaling. As summarized in Figure 5, we should work to balance insulin/insulin-like growth factor signaling to manage the number of VSELs in adult tissues. If insulin/insulin-like growth factor signaling predominates, it will prematurely deplete VSELs from adult tissues, leading to a shorter lifespan and an increased risk of malignancies. Conversely, with a proper diet and regular physical activity, we can anticipate healthy aging and a reduced risk of cancer.

Our story describing VSELs aligns with a recent excellent paper (Cancedda and Mastrogiacomo, 2024) that presents the viewpoint that in mammals, from birth through adulthood, epiblast-like pluripotent cells are maintained in the bone marrow and likely in the gonads as a very small percentage of the total cell population. These pluripotent cells are characterized by their small size, large nucleus, and limited cytoplasm. They express stage-specific embryonic antigens (SSEA) and pluripotent genes found in early embryos, including Oct3/4, Sox2, Klf4, Nanog, and c-Myc. As early developmental embryonic stem cells, they possess the ability for self-renewal and the potential to differentiate into cell lineages derived from all three germ layers: ectoderm, mesoderm, and endoderm. Therefore, they can be classified as adult pluripotent stem cells. However, unlike embryonic stem cells, they have lost their teratogenic and tumorigenic properties after being grafted into immunodeficient mice. These adult pluripotent stem cells are quiescent and self-renew at a very low rate in postnatal tissues.
