# Supplementary material for: Very small embryonic-like stem cells (VSELs) on the way for potential applications in regenerative medicine
Source: Front Bioeng Biotechnol. 2025 Mar 7;13:1564964. doi: 10.3389/fbioe.2025.1564964 (PMC11926153; doi:10.3389/fbioe.2025.1564964)
Supplement: Supplementary file 1 [file DataSheet1.pdf]

Supplementary Table I

Selected papers reporting presence of VSELs in postnatal tissues

A. *Papers where investigators* ***were able to successful isolate small cells with VSELs*** *phenotype –* ***one selected publication per group excluding papers where I am a co-author***
Howell JC, *et al.* Pluripotent stem cells identified in multiple murine tissues. Annals of the New York Academy of Sciences. 2003; 996:158-173
McGuckin C, *et al.* Culture of embryonic-like stem cells from human umbilical cord blood and onward differentiation to neural cells in vitro. Nature Protocols. 2008; 3:1046-1055
Halasa M, *et al.* An efficient two-step method to purify very small embryonic-like (vsel) stem cells from umbilical cord blood (ucb). Folia Histochemica et Cytobiologica. 2008;46:239-243
Dyce PW, *et al.* In vitro and in vivo germ line potential of stem cells derived from newborn mouse skin. PLoSOne. 2011;6:e20339.
Wu JH, *et al.* Characterization of rat very small embryonic-like stem cells and cardiac repair after cell transplantation for myocardial infarction. Stem Cells Dev. 2012; 21(8):1367-79.
Iskovich S, *et al.* Elutriated stem cells derived from the adult bone marrow differentiate into insulin-producing cells in vivo and reverse chemical diabetes. Stem Cells Dev. 2012; 21:86–96.
Shirazi R, *et al.* BMP4 can generate primordial germ cells from bone-marrow-derived pluripotent stem cells. Cell Biol Int. 2012; 36(12):1185-93.
Kassmer SH, *et al.* Very small embryonic-like stem cells from the murine bone marrow differentiate into epithelial cells of the lung. Stem Cells. 2013;31: 2759-66.
Igura K, *et al.* Identification of small juvenile stem cells in aged bone marrow and their therapeutic potential for repair of the ischemic heart. Am J Physiol Heart Circ Physiol. 2013; 305(9):H1354-62.
Havens AM, *et al.* Human and murine very small embryonic-like cells represent multipotent tissue progenitors, in vitro and in vivo. Stem Cells and Development. 2014; 23:689-701
Chang YJ, *et al.* Recovery of cd45(-)/lin(-)/ssea-4(+) very small embryonic-like stem cells by cord blood bank standard operating procedures. Cytotherapy. 2014; 16:560-565
Lee SJ, *et al.* Adult stem cells from the hyaluronic acid-rich node and duct system differentiate into neuronal cells and repair brain injury. Stem Cells Dev. 2014; 23(23):2831-40.
Hwang S, *et al.* Nonmarrow hematopoiesis occurs in a hyaluronic-acid-rich node and duct system in mice. Stem Cells Dev. 2014; 23(21):2661-71.
Abouzaripour M, *et al.* Intravenous transplantation of very small embryonic like stem cells in treatment of diabetes mellitus. Avicenna J Med Biotechnol. 2015; 7(1):22-31.
Sriraman K, *et al.* Mouse Ovarian Very Small Embryonic-Like Stem Cells Resist Chemotherapy and Retain Ability to Initiate Oocyte-Specific Differentiation. Reprod Sci. 2015; 22:884–903.
Guerin CL, *et al.* Bone-marrow-derived very small embryonic-like stem cells in patients with critical leg ischaemia: Evidence of vasculogenic potential. Thrombosis and Haemostasis. 2015; 113:1084-1094
Chen ZH, *et al.* Hepatic regenerative potential of mouse bone marrow very small embryonic-like stem cells. Journal of Cellular Physiology. 2015; 230:1852-1861
Shaikh A, *et al.* Molecular and phenotypic characterization of CD133 and SSEA4 enriched very small embryonic-like stem cells in human cord blood. Leukemia. 2015; 29(9):1909-17.
Lo Sicco C, *et al.* Identification of a New Cell Population Constitutively Circulating in Healthy Conditions and Endowed with a Homing Ability Toward Injured Sites. Sci Rep. 2015; 5:16574.
Nakatsuka R, *et al.* Identification and characterization of lineage(-)cd45(-)sca-1(+) vsel phenotypic cells residing in adult mouse bone tissue. Stem Cells and Development. 2016; 25:27-42
Golipoor Z, *et al.* Migration of Bone Marrow-Derived Very Small Embryonic-Like Stem Cells toward An Injured Spinal Cord. Cell J. 2016; 17(4):639-47
Zhang S, *et al.* HIF-2α and Oct4 have synergistic effects on survival and myocardial repair of very small embryonic-like mesenchymal stem cells in infarcted hearts. Cell Death Dis. 2017; 8(1):e2548.
Monti M, *et al.* A Novel Method for Isolation of Pluripotent Stem Cells from Human Umbilical Cord Blood. Stem Cells Dev. 2017; 26(17):1258-1269.
Lahlil R, *et al.* VSELs Maintain their Pluripotency and Competence to Differentiate after Enhanced Ex Vivo Expansion. Stem Cell Rev. 2018; 14(4):510-524.
Gounari E, *et al.* Isolation of a novel embryonic stem cell cord blood-derived population with in vitro hematopoietic capacity in the presence of Wharton's jelly-derived mesenchymal stromal cells. Cytotherapy. 2019; 21(2):246-259.
Eljaszewicz A, *et al.* Very Small Embryonic-Like Stem Cells, Endothelial Progenitor Cells, and Different Monocyte Subsets Are Effectively Mobilized in Acute Lymphoblastic Leukemia Patients after G-CSF Treatment. Stem Cells Int. 2018; 2018:1943980.
Makar T, *et al.* A subset of mobilized human hematopoietic stem cells express germ layer lineage genes which can be modulated by culture conditions. Stem Cell Res Ther. 2018; 9(1):127.
Kim Y, *et al.* Small hypoxia-primed mesenchymal stem cells attenuate graft-versus-host disease. Leukemia. 2018; 32(12):2672-2684.
Virant-Klun I, *et al.* Similar Population of CD133+ and DDX4+ VSEL-Like Stem Cells Sorted from Human Embryonic Stem Cell, Ovarian, and Ovarian Cancer Ascites Cell Cultures: The Real Embryonic Stem Cells? Cells. 2019; 8(7). pii: E706
Kuruca SE, *et al.*. Characterization and Isolation of Very Small Embryonic-like (VSEL) Stem Cells Obtained from Various Human Hematopoietic Cell Sources. Stem Cell Rev. 2019, 15(5):730-742.<br>Lahlil R, Aries A, Scrofani M, Zanetti C, Hennequin D, Drénou B. Stem Cell Responsiveness to Imatinib in Chronic Myeloid Leukemia. Int J Mol Sci. 2023 Nov 23;24(23):16671.<br>Filidou E, Kandilogiannakis L, Tarapatzi G, Spathakis M, Su C, Rai A, Greening DW, Arvanitidis K, Paspaliaris V, Kolios G. A Simplified and Effective Approach for the Isolation of Small Pluripotent Stem Cells Derived from Human Peripheral Blood.<br>Biomedicines. 2023 Mar 5;11(3):787.

Kamil G, Karolina S, Aleksandra S, Filip B, Marta P, Artur B, Marcin M. Alterations in Stem Cell Populations in IGF-1 Deficient Pediatric Patients Subjected to Mecasermin (Increlex) Treatment. Stem Cell Rev Rep. 2023 Feb;19(2):392-405.

Jamiołkowska-Sztabkowska M, Grubczak K, Starosz A, Kretowska-Grunwald A, Kretowska M, Parfienowicz Z, Moniuszko M, Bossowski A, Głowińska-Olszewska B. Circulating Hematopoietic (HSC) and Very-Small Embryonic like (VSEL) Stem Cells in Newly Diagnosed Childhood Diabetes type 1 - Novel Parameters of Beta Cell Destruction/Regeneration Balance and Possible Prognostic Factors of Future Disease Course. Stem Cell Rev Rep. 2022 Jun;18(5):1657-1667.

Hénon P. Key Success Factors for Regenerative Medicine in Acquired Heart Diseases.Stem Cell Rev Rep. 2020 Jun;16(3):441-458.

Dąbkowski K, Łabędź-Masłowska A, Dołęgowska B, Safranow K, Budkowska M, Zuba-Surma E, Starzyńska T. Evidence of Stem Cells Mobilization in the Blood of Patients with Pancreatitis: A Potential Link with Disease Severity. Stem Cells Int. 2022 Jul 8;2022:5395248.

Hénon P, Kowalczyk M, Aries A, Vignon C, Trébuchet G, Lahlil R. Industrialized GMP Production of CD34+ Cells (ProtheraCytes®) at Clinical Scale for Treatment of Ischemic Cardiac Diseases Is Feasible and Safe. Stem Cell Rev Rep. 2022 Jun;18(5):1614-1626.

Guenther R, Dreschers S, Maassen J, Reibert D, Skazik-Voogt C, Gutermuth A. The Treasury of Wharton's Jelly. Stem Cell Rev Rep. 2022 Jun;18(5):1627-1638.

Ebrahim N, Al Saihati HA, Shaman A, Dessouky AA, Farid AS, Hussien NI, Mostafa O, Seleem Y, Sabry D, Saad AS, Emam HT, Hassouna A, Badr OAM, Saffaf BA, Forsyth NR, Salim RF. Bone marrow-derived mesenchymal stem cells combined with gonadotropin therapy restore postnatal oogenesis of chemo-ablated ovaries in rats via enhancing very small embryonic-like stem cells. Stem Cell Res Ther. 2021 Sep 27;12(1):517.

Wang CY, Yu GT, Gao C, Chen J, Li QL, Zhang L, Wu M, Sun ZJ, Li LY. Genome-Wide Enhancer Analysis Reveals the Role of AP-1 Transcription Factor in Head and Neck Squamous Cell Carcinoma. Front Mol Biosci. 2021 Aug 2;8:701531.

Barati M, Akhondi M, Mousavi NS, Haghparast N, Ghodsi A, Baharvand H, Ebrahimi M, Hassani SN. Pluripotent Stem Cells: Cancer Study, Therapy, and Vaccination. Stem Cell Rev Rep. 2021 Dec;17(6):1975-1992.

Sun X, Li H, Zhu Y, Xu P, Zuo Q, Li B, Gu X. 5-Azacytidine-Induced Cardiomyocyte Differentiation of Very Small Embryonic-Like Stem Cells. Stem Cells Int. 2020 Sep 8;2020:5162350.

Haj-Mirzaian A, Khosravi A, Haj-Mirzaian A, Rahbar A, Ramezanzadeh K, Nikbakhsh R, Pirri F, Talari B, Ghesmati M, Nikbakhsh R, Dehpour AR. The potential role of very small embryonic-like stem cells in the neuroinflammation induced by social isolation stress: Introduction of a new paradigm. Brain Res Bull. 2020 Oct;163:21-30.

B. *Papers where investigators encountered technical problems to isolate small cells with VSELs phenotype – published more than 10 tears ago* ***(we addressed their mistakes in VSELs isolation strategies point by point as published in Leukemia. 2014; 28(3):473-84. and Stem Cells Dev. 2014; 23(7):702-13.)***
Danova-Alt R, *et al.* Very small embryonic-like stem cells purified from umbilical cord blood lack stem cell characteristics. PLoS One. 2012; 7(4):e34899.
Miyanishi M, *et al.* Do pluripotent stem cells exist in adult mice as very small embryonic stem cells? Stem Cell Reports. 2013; 1(2):198-208.
Szade K, *et al.* Murine bone marrow Lin$^-$Sca$^-$1$^+$CD45$^-$ very small embryonic-like (VSEL) cells are heterogeneous population lacking Oct-4A expression. PLoS One. 2013; 8(5):e63329.
